# Family-based genome-wide association study for simulated data of Framingham Heart Study

**DOI:** 10.1186/1753-6561-3-s7-s124

**Published:** 2009-12-15

**Authors:** Hongyan Xu, George Mathew, Varghese George

**Affiliations:** 1 Department of Biostatistics, Medical College of Georgia, 1469 Laney Walker Boulevard, Augusta, Georgia 30912-4900, USA; 2 Department of Mathematics, Missouri State University, 901 South National Avenue, Springfield, Missouri 65897, USA

## Abstract

Genome-wide association studies (GWAS) have quickly become the norm in dissecting the genetic basis of complex diseases. Family-based association approaches have the advantages of being robust to possible hidden population structure in samples. Most of these methods were developed with limited markers. Their applicability and performance for GWAS need to be examined. In this report, we evaluated the properties of the family-based association method implemented by ASSOC in the S.A.G.E package using the simulated data sets for the Framingham Heart Study, and found that ASSOC is a highly useful tool for GWAS.

## Background

Genome-wide association studies (GWAS) are gaining popularity in genetic analysis of complex traits with the development of genotyping technology at the genome level. With genetic information at millions of single-nucleotide polymorphisms (SNPs) and other genetic markers, such studies offer great opportunities and also present challenges in developing appropriate statistical analysis methods. The data distributed by Genetic Analysis Workshop 16 provides a great opportunity to examine the strengths and limitations of current statistical methods for GWAS.

Many GWAS have been reported in the literature and many more are being performed. Most of them are population-based association studies and use designs such as case-control studies. Such designs have the advantages that samples are easy to ascertain and that results have relatively high power when analyses are carried out properly. However, it has been shown that population-based approaches, such as case-control studies, can produce spurious associations in the presence of population substructure, especially in large-scale studies at the genomic level [1,2]. An alternative approach is to use the family-based association methods, such as the transmission-disequilibrium test [3], the family-based association test (FBAT) [4], and a regression method [5] implemented by ASSOC in the S.A.G.E. package. These methods are robust to population substructure and other cryptic relatedness in the samples. However, these methods have been proposed in the era with only a few genetic markers and are intended mostly for candidate-gene studies. Their applicability and performance for GWAS has not been examined. The simulated data set of the Framingham Heart Study (FHS) provides both the family structure and the genotype information at genome-wide SNPs. The underlying simulation models are also provided. It is the purpose of this study to evaluate the performance of the family-based association method implemented by ASSOC in analyzing GWAS.

## Methods

ASSOC implements a regression method that tests for association between a continuous trait and one or more covariates, including genetic markers from extended family data, and accounts for familial correlations [5]. The program estimates the parameters of a baseline model and those of alternate models that include specific sets of covariates. A likelihood-ratio test is then performed to evaluate the significance of the covariates.

The FHS simulated data set includes 6,476 subjects with both phenotype and genotype data in 942 pedigrees of up to three generations and 188 singletons. We excluded the singletons from our analysis because they do not have family information. The simulated data contain 200 replicates of the phenotypes. We used the phenotype data from the first replicate. Pedigree files were constructed from the supplied 'triplet_sim' file and merged with phenotype and genotype data. In addition to each of the SNPs, we included subject's age, smoking status, diet, and lipid-lowering drug usage as covariates. We kept all family members in the analysis because ASSOC could account for the familial correlations. We chose to test the association of HDL with all the makers from chromosome 19 in the 50 k panel and 500 k panel because there are two major genes for HDL on chromosome 19. In all, there are 1,639 markers in the 50 k panel and 6,350 markers on 500 k panel on chromosome 19. We assumed an additive model for all the markers. The program was run in a batch mode so that it would test for the association of the SNPs successively in one run. From the simulation model, there are five major genes for HDL. Two were on chromosome 19, and the other three were from chromosomes 8, 9, and 15, respectively. The chromosome locations of the five major genes were given in Table 1. We tested the association of HDL with all the five major genes. There are a total of 1,000 polygenes for HDL, among which 15 are on chromosome 19.

**Table 1:** Test results of the 5 SNPs from 500 k panel for major gene effects of HDL

Model	Location	Intercept	Diet	Sex	Rx^a^	Age	Smoke^b^	SNP	SE	*p*-Value
rs10820738	9q31.1	68.694	0.028	13.565	1.486	0.019	0.714	4.524	0.518	3.20 × 10^-18^
rs3200218	8p22	68.680	0.027	13.552	1.420	0.020	0.664	-1.671	0.319	1.63 × 10^-7^
rs8035006	15q21	68.684	0.023	13.527	1.474	0.020	0.647	0.831	0.284	0.003
rs8192719	19q13.2	68.689	0.021	13.524	1.470	0.020	0.666	-0.821	0.306	0.007
rs8103444	19113.2	68.686	0.021	13.533	1.452	0.019	0.660	0.629	0.305	0.039

^a^Rx, lipid lowering drug use

^b^Smoke, smoking status

## Results

We performed association tests of all 1,639 markers on chromosome 19 with simulated HDL data. Out of 1,639 markers, the tests of 18 markers were significant at the 0.01 level. Because the HDL data were simulated using the markers in the 500 k panel and not the markers in the 50 k panel, and since the markers in the two panels are not close, we assume that the markers in the 50 k panel did not contribute to the HDL phenotype. The significance result reflects false positives. Therefore, our results give an empirical type I error probability of 0.011, which agrees well with the nominal level of 0.01.

We further tested the association of the five SNPs representing the major genes with HDL (Table 1). They were all significant at 0.05 level regardless of the mode of inheritance and heritability. The true genetic models are additive for rs8103444, rs8035006, and rs8192719, and dominant for rs10820738 and rsrs3200218. However, if more stringent significance level were used for genome-wide studies, only two markers, rs10820738 and rs3200218, were found significant at 10^-6 ^level. Table 1 also gives the estimated effect size for the five SNPs. SNP rs10820738 has the largest effect size, which is consistent with the simulation model in which it has the largest heritability.

**Figure 1:**
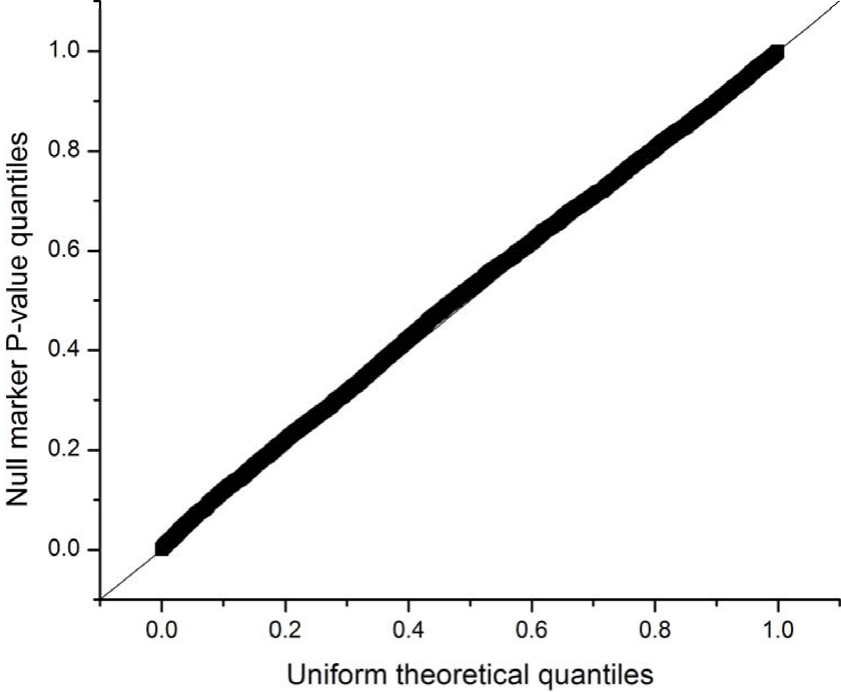
**Quantile-quantile plot of the *p*-values from null markers**.

We then tested all 6,350 SNP markers on chromosome 19 in the 500 k panel for association with the simulated HDL data. Among the 15 markers that have polygenic effects on chromosome 19, association tests at six markers were significant at the 0.05 level (Table 2). The marker positions in base pairs are also given in Table 2. The pairwise LD was relatively low, with a maximum *r*^2 ^of 0.003 as computed with Haploview [6]. A total of 395 tests were significant at 0.05 level for all the 6,350 markers tested on chromosome 19. Excluding the two major genes and six polygenes that are truly associated with the trait, this gives an estimated empirical type I error rate of 0.06. Figure 1 gives the quantile-quantile plot of the *p*-values for all the null markers. The observed distribution fits well with the expected uniform distribution and there is no major inflation of type I error. Among the top 50 SNPs ranked by *p*-values, four SNPs are true findings with polygenic effects.

**Table 2:** Test results among polygenes affecting HDL on chromosome 19

Model	Position	Intercept	Diet	Sex	Rx^a^	Age	Smoke^b^	SNP	SE	*p*-Value
rs10420985	62,077,748	68.693	0.017	13.519	1.508	0.020	0.656	-1.693	0.457	0.000214
rs8182590	13,282,550	68.691	0.029	13.542	1.478	0.019	0.686	-0.973	0.263	0.000222
rs35150881	2,572,193	68.689	0.023	13.537	1.449	0.019	0.663	-1.727	0.550	0.0017
rs8107007	33,248,124	68.691	0.022	13.548	1.454	0.020	0.651	1.683	0.537	0.001723
rs10403702	44,840,424	68.692	0.021	13.532	1.491	0.020	0.647	5.564	2.199	0.011418
rs7251886	46,880,615	68.687	0.020	13.537	1.501	0.020	0.669	-0.666	0.266	0.012645
rs11673050	35,535,929	68.690	0.019	13.530	1.473	0.020	0.659	-0.505	0.279	0.069854
rs2277987	8,458,273	68.689	0.021	13.549	1.480	0.020	0.658	0.496	0.289	0.08626
rs16989305	49,579,243	68.689	0.023	13.540	1.482	0.020	0.666	4.904	3.111	0.115021
rs16966229	36,924,150	68.687	0.021	13.530	1.461	0.020	0.660	-2.165	1.381	0.117801
rs17620029	24,146,152	68.690	0.019	13.539	1.465	0.020	0.652	-0.390	0.369	0.291036
rs3786501	49,691,015	68.691	0.021	13.536	1.473	0.020	0.656	0.217	0.314	0.490909
rs11085876	13,947,513	68.689	0.020	13.540	1.467	0.020	0.655	-0.164	0.273	0.547852
rs17716486	34,455,727	68.690	0.020	13.539	1.478	0.020	0.655	-0.204	0.376	0.586957
rs599458	20,792,763	68.689	0.021	13.538	1.473	0.020	0.657	0.082	0.454	0.856762

^a^Rx, lipid lowering drug use

^b^Smoke, smoking status

We also applied FBAT to test the association of HDL with all 6,350 SNPs on chromosome 19. A total of 325 tests are significant at 0.05 level. However, only two polygenes are true positives (rs10420985 and rs599458, with *p*-values 0.004408 and 0.028563, respectively). None of the major genes was significant with FBAT. Nonetheless, the type I error rate seems well controlled.

## Discussion

Family-based association methods are appealing alternatives for the population-based case-control design because they are robust to population stratification in the samples. Several such methods have been proposed. However, they were all proposed before the current genomic era. As the norm of the field moves to GWAS, the performance and applicability of these methods need to be examined for GWAS. In this report, we examined the performance of a regression-based method [5], implemented in the program ASSOC in the software package S.A.G.E., using the simulated HDL data in the Framingham Heart Study. Based on the results of the tests with the markers on chromosome 19 in the 50 k panel, we found that ASSOC gives the correct type I error rate. When applied to the markers on chromosome 19 in the 500 k panel (tests performed at 0.05 level), the empirical type I error rate was 0.06, which is slightly inflated. The reason could be that there is linkage disequilibrium between the markers in the causal genes and markers close by, and when we estimated type I error rate, we only excluded the markers in the causal genes with either major or polygenic effects and not those markers in linkage disequilibrium. Therefore, as a general conclusion, ASSOC gives a more-or-less the correct type I error rate, and hence is a valid test for GWAS.

In our analysis, ASSOC detected all five major genes and six of the 15 polygenes for HDL on chromosome 19. In contrast, FBAT detected only two of the 15 polygenes and none of the major genes on chromosome 19. It should be noted that the data may be too limited to give a reliable estimation of the power. However, it is encouraging to see that ASSOC could detect one of the polygenes, rs10403702, whose minor allele frequency is only 0.35%. Current association studies generally focus on common SNPs (e.g., SNPs with minor allele frequency > 5%) based on the common disease, common variants hypothesis [7-10]. The other reason is that the statistical power may not be sufficient for rare SNPs when the sample size is limited. However, recent development in genotyping technology allows efficient genotyping in large samples and there is a call for shifting the paradigm of association studies to rare SNPs because it may be more effective to discover susceptibility genes for common diseases [11].

In conclusion, the method implemented in ASSOC provides a valid association test for family-based data and is reasonably powerful approach to be applied in GWAS. However, it should be noted that it is also a rather slow method. In our analysis, it took around 10 minutes to test one marker in our Windows-based workstation with 2.13 GHz CPU. It will take substantial amount of time to perform the test for millions of markers in GWAS. Parallel computing would be the solution.

## Conclusion

Using the simulated data for the Framingham Heart Study, we found the family-based regression method George et al. [5] implemented in ASSOC in the S.A.G.E. software is applicable to GWAS. It provides the correct type I error rate and reasonable power. However, this method is computationally time-consuming.

## List of abbreviations used

FBAT: Family-based association test; FHS: Framingham Heart Study; GWAS: Genome-wide association studies; SNP: Single-nucleotide polymorphism.

## Competing interests

The authors declare that they have no competing interests.

## Authors' contributions

HX conceived of the study, performed the analysis, and drafted the manuscript. GM participated in the analysis and helped to draft the manuscript. VG participated in the design and coordination of the study. All authors read and approved the final manuscript.
